# The complement system in neurodegenerative and inflammatory diseases of the central nervous system

**DOI:** 10.3389/fneur.2024.1396520

**Published:** 2024-07-03

**Authors:** Luciana Negro-Demontel, Adam F. Maleki, Daniel S. Reich, Claudia Kemper

**Affiliations:** ^1^National Heart, Lung, and Blood Institute (NHLBI), National Institutes of Health (NIH), Complement and Inflammation Research Section (CIRS), Bethesda, MD, United States; ^2^Department of Histology and Embryology, Faculty of Medicine, UDELAR, Montevideo, Uruguay; ^3^Neuroinflammation and Gene Therapy Laboratory, Institut Pasteur de Montevideo, Montevideo, Uruguay; ^4^Translational Neuroradiology Section, National Institute of Neurological Disorders and Stroke (NINDS), NIH, Bethesda, MD, United States

**Keywords:** complement, complosome, central nervous system, neuroinflammation, neurodegeneration, brain, development

## Abstract

Neurodegenerative and neuroinflammatory diseases, including Alzheimer’s disease, Parkinson’s disease, and multiple sclerosis, affect millions of people globally. As aging is a major risk factor for neurodegenerative diseases, the continuous increase in the elderly population across Western societies is also associated with a rising prevalence of these debilitating conditions. The complement system, a crucial component of the innate immune response, has gained increasing attention for its multifaceted involvement in the normal development of the central nervous system (CNS) and the brain but also as a pathogenic driver in several neuroinflammatory disease states. Although complement is generally understood as a liver-derived and blood or interstitial fluid operative system protecting against bloodborne pathogens or threats, recent research, particularly on the role of complement in the healthy and diseased CNS, has demonstrated the importance of locally produced and activated complement components. Here, we provide a succinct overview over the known beneficial and pathological roles of complement in the CNS with focus on local sources of complement, including a discussion on the potential importance of the recently discovered intracellularly active complement system for CNS biology and on infection-triggered neurodegeneration.

## Introduction

Aging is inevitably accompanied by physiological changes in cellular behavior that overall result in decline in homeostasis and function and, eventually, cell death. Age-related perturbations in cellular activities and neuronal cell loss in the central nervous system (CNS) are major underlying causes for neurodegenerative diseases, including Alzheimer’s disease (AD), amyotrophic lateral sclerosis (ALS), Huntington’s disease (HD), and Parkinson’s disease (PD) (1). Furthermore, neuroinflammation, defined as an inflammatory response within the brain and/or spinal cord, often cumulates in neuronal loss and neurodegeneration, as seen in the autoimmune inflammatory condition multiple sclerosis (MS) (2). Although some risk factors and disease-driving molecular mechanisms for neurodegenerative or neuroinflammatory diseases have been identified, generally, disease pathologies are not well understood. This, in combination with the fact that targeting the CNS therapeutically remains difficult due to the highly effective function of the blood–brain barrier (BBB) as a selective CNS entry guardian (3), contributes to a lack of effective drugs targeting primary or secondary neurodegeneration.

The complement system, best known as a key arm of innate immunity, has gained attention as a major player in healthy CNS biology based on its contributions to normal neuronal development, but also for its involvement in inflammatory processes within the CNS. Studies have revealed dysregulation of complement activation in various neurodegenerative and inflammatory conditions, including AD and MS (4, 5). Interestingly, both beneficial and pathological activities of complement in the brain or spinal cord are majorly dependent on locally produced complement with limited involvement from the liver-derived circulating complement (6). This observation is in line with recent adjustments in our understanding of the complement system. Initially, complement was thought to be a circulation- or vessel-operative system with only a simple role in mediating the detection and removal of bloodborne pathogens. Today, we acknowledge that the complement system is operative at different locations that span the vasculature, the extracellular space in tissues where it is critical in mediating protective tissue immunity (7, 8), and within cells where it regulates basic cellular processes (9). The functional reach of complement allows it to directly modulate innate and adaptive immune responses and the behavior of non-immune cells, both during homeostasis and in response to danger-associated molecular patterns (DAMPs) and other noxious triggers. Furthermore, complement has emerged as a key mediator of tissue homeostasis, repair, and regeneration and as such is also involved in the molecular pathways underlying resolution of CNS inflammation and remyelination of neurons after myelin sheath loss, for example, in MS.

Here, we will give a condensed overview of the known sources and roles of complement components in normal CNS function, such as neuronal development and nerve pruning, as well as in disease pathologies contributing to neurodegenerative or neuroinflammatory pathogeneses, and processes that may aid in the resolution or repair of CNS tissue injury. Not included here are the emerging roles of complement in pathologies of peripheral nerves or neuromuscular junctions, such as Guillain-Barré syndrome, and myasthenia gravis (MG), and others (1). Of note though, MG is currently the sole neurodegenerative disease successfully targeted by an anti-complement drug (10, 11). We will conclude with a summary on emerging areas of new complement locations and activities that we suggest could be important in CNS pathologies, such as the intracellularly active complement system and its tight association with the control of single cell physiology and a potential connection between viral infections, complement, and neuroinflammation. This review is not intended to be a comprehensive overview on complement in the CNS but rather a succinct starting point for newcomers to the field, and a perspective on novel developments in the complement field to watch. We refer readers seeking in-depth insights into the roles of complement across specific CNS-associated diseases to excellent recent reviews by respective experts in the field such as (2, 4–6).

## The complement system

The complement system was discovered by Jules Bordet over a century ago (12) and consists of over 50 proteins that are either present in circulation in the blood and interstitial fluids (the core components and fluid-phase regulators), anchored to cell-surface membranes (receptors and membrane-bound regulators) or present within cells in subcellular compartments (all components) (13, 14). The following provides an abbreviated overview over the currently known locations and functions of the contemporary complement system.

### Extracellular complement—activation, function, and regulation

**Figure 1:**
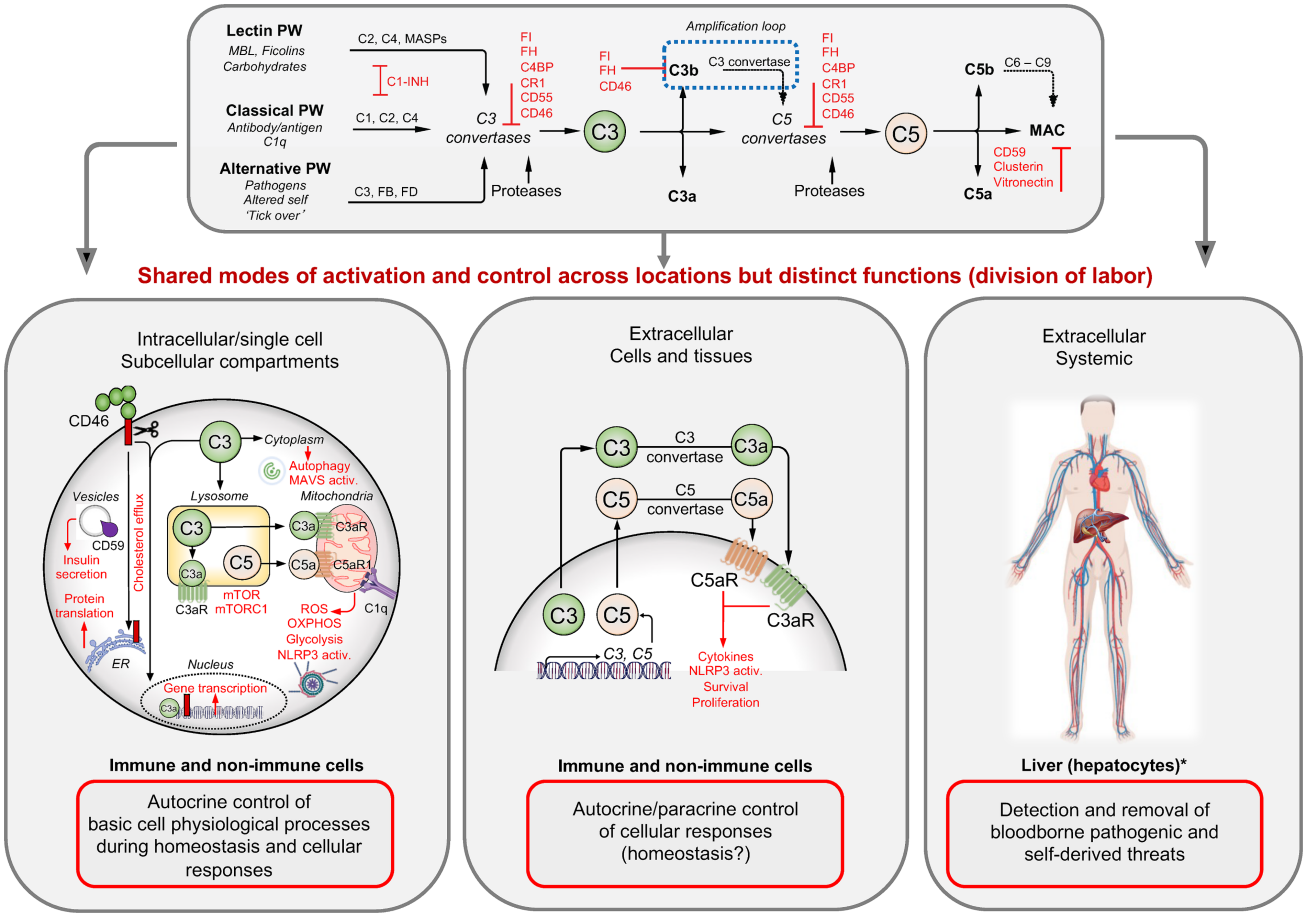
Complement activation, regulation, and functions. Three pathways lead to the formation of C3 and C5 convertases that then cleavage-activate C3 into C3a and C3b, and C5 into C5a and C5b. Assembly of C5b with C6–C9 induces the insertion of the membrane attack complex (MAC) into target membranes while C3b/iC3b opsonizes and tags targets for phagocytic uptake and the anaphylatoxins C3a and C5a mediate the classical inflammatory reactions. The system is controlled by several fluid-phase and membrane-bound complement regulators (red). In circulation, this system detects and removes invading pathogens and noxious antigens. In tissues, C3 and C5 are secreted by immune and non-immune cells, and the extracellularly generated C3 and C5 activation fragments induce cell-specific responses in an autocrine and/or paracrine manner. Intracellularly active complement (the complosome) operates across a broad range of cell populations and at different subcellular locations, where they majorly control basic physiological processes, often in direct crosstalk with other intracellular danger-sensing systems. C1-INH, C1 esterase inhibitor; C3aR, C3a receptor; C5aR, C5a receptor; C4BP, C4b-binding protein; CR1, complement receptor 1; ETC, electron transport chain; FB, factor B; FD, factor D; FH, factor H; FI, factor I; MASP, MBL-associated serine protease; MAVS, mitochondrial antiviral signaling protein; MBL, mannose-binding lectin; mTOR, mechanistic target of rapamycin; OXPHOS, oxidative phosphorylation; RIG-I, retinoic acid-inducible gene I; ROS, reactive oxygen species. Created with BioRender.com.

The complement components C3 and C5 are considered the major effector molecules of the complement system. They are produced and secreted by the liver in pro-enzymatic forms and circulate through the blood and lymph. C3 activation into bioactive C3a and C3b is initiated when one or several complement activation pathways are triggered by the binding of their respective pattern recognition receptor (PRR) components to pathogen or danger-associated molecular patterns (PAMPs or DAMPs) (Figure 1) (13). The classical pathway (CP) is initiated when C1q binds to antibody–antigen complexes on the surface of pathogens or to DAMPs exposed by altered self, for example, apoptotic cells. The lectin pathway (LP) is triggered by the mannose-binding lectin MBL, collectins, or ficolin-mediated detection of microbial oligosaccharides and acetylated residues (13). The alternative pathway (AP) is continuously activated by tonic hydrolysis of the internal C3 thioester bond into C3(H2O) and then further amplified through contact with pathogenic, microbial, or noxious self-derived antigens (13). All three pathways culminate in the formation of the CP/LP and AP C3 convertases (C4bC2b and C3bBb) that then cleavage-activate C3 into C3a and C3b. The increased deposition of C3b to target surfaces in the vicinity of C3 convertases fosters immediate formation of the CP/LP, and AP C5 convertases (C4bC2bC3b and C3bBbC3b) which cleave C5 into C5a and C5b.

C3b opsonizes microbes and dangerously altered host cells and provides a signal for phagocytic uptake by scavenger cells (15) and C5b combines with serum C6 to C9 to form the membrane attack complex (MAC), which inserts into target membranes and mediates direct lytic killing of pathogens or infected or stressed host cells (13). The G protein-coupled receptors (GPCRs) for the anaphylatoxins C3a and C5a, C3aR and C5aR1 and C5aR2, are broadly expressed by host immune and non-immune cells (13). Their activation is key to inducing the classic inflammatory reaction, which involves activation of the endothelium leading to adherence and tissue influx of immune cells, smooth muscle cell contraction, and the migration and activation of innate immune cells to the site of infection or insult (13). Overall, target opsonization, lytic killing of pathogens, and innate immune cell activation represent the canonical complement functions and functional deficiencies in complement effector components cause recurrent infections (16, 17). However, complement deficiencies are also associated with autoimmune diseases such as systemic lupus erythematosus (SLE) (18, 19) because complement also detects and removes self-derived dangers such as immune complexes (ICs) and apoptotic or stressed cells (15, 19). In fact, it is now well understood that complement is an active participant in the repair processes underlying the regeneration of tissues after immune-response-associated tissue pathology or after injury and trauma (15).

A continuously better understanding of the structures and functions of the single complement components revealed that the complement system plays also central roles in adaptive immunity. For example, signals mediated by the complement receptor 2 (CR2, CD21) provide important co-stimulatory input during B cell activation (20, 21). Similarly, cell-intrinsic engagement of the complement regulator and receptor CD46 during T cell receptor engagement (TCR) on CD4 and CD8 T cells provides co-stimulatory signals that are required for T helper type 1 (Th1) and cytotoxic T lymphocyte (CTL) responses, respectively (7, 22–24). Importantly, the engagement of complement receptors on immune cells in tissues is largely independent of liver-produced complement components, but majorly driven by immune cell-produced and local complement activation (Figure 1). For example, C3 and C5 are secreted by activated antigen presenting cells (APCs) and/or by T cells during the cognate APC–T cell interaction and cleavage-activated in the extracellular space by C3/C5 convertases, which form via immune cell-provided Factor (F) D and FB (25–27). The anaphylatoxins generated locally then engage their respective receptors on cells in the vicinity in an autocrine and/or paracrine manner and induce the expression of co-stimulatory molecules on APC and T cell, cell proliferation, and the production of cytokines—with pathological decreases or increases in immune cell-provided complement causing infections or autoimmunity, respectively (8).

Once activated, complement components latch onto surfaces indiscriminately (15). To prevent detrimental tissue pathologies due to unwanted or chronic complement activation, the system is tightly controlled by a range of fluid-phase and cell-expressed regulators (Figure 1). These operate on three basic levels: prevention of C3b and C4b deposition on host cells in the first place by inactivating C3b and C4b; active dissociation of host cell surface formed C3/C5 convertases; and prevention of MAC insertion.

### Intracellular complement—activation, function, and regulation

Recent work has revealed, unexpectedly, that the location of complement activities is not confined to the extracellular space, but that complement is also active within cells (7) (Figure 1). Although the intracellularly operating complement [which was coined “the complosome” (28)] was initially discovered in human CD4 T cells (7), the complosome has, by now, been detected in a broad range of immune and non-immune cell populations. There have been recent excellent in-depth reviews on the roles of intracellular complement activities in health and disease (8, 29–32), and we will therefore provide a short summary only about the system here.

Complement proteins constituting the complosome are encoded by the same genes that generate complement components in circulation, they are often expressed tonically by non-hepatocytes and can be modulated by incoming environmental cues or cell stimulation (8, 33–35). Although in most cells, components of the complosome originate from cell-intrinsic expression, they can be sourced from the extracellular milieu or the cell surface to become part of the intracellularly active complement system. Similar to complement in circulation, intracellular C3 and C5 can be cleavage-activated by specific proteases or by intracellular C3 and C5 convertases that form beneath the plasma membrane and on the surface of subcellular compartments (35–37). Furthermore, intracellular C3 activation in macrophages is negatively controlled by cell-intrinsic FH (38), indicating that the complosome is subject to regulation by “classic” complement regulators. Of note, cell autonomous FH activities emerge as a particular area of interest as FH not only controls complosome activation but also serves non-canonical roles in epithelial cells where it may directly control nuclear factor kappa B (NF-κB) nuclear translocation and activities (39, 40). C3 and C5, and/or their activation fragments and receptors, C1q, and some regulators have been detected in the nucleus, cytoplasm, lysosomes, endoplasmic reticulum, autophagosomes, and/or on the outer membrane of mitochondria. Importantly, the distinct intracellular location of complement components provides them with the capacity to populate a discrete functional niche: the complosome serves roles that associated with the direct control of basic cell physiological processes, including gene transcription, cell metabolism, vesicular transport and secretion and autophagy (41–44), as well intracellular danger sensing in cooperation with the nucleotide-binding domain, leucine-rich–containing family, pyrin domain–containing-3 (NLRP3) inflammasome, toll-like receptors (TLRs), and mitochondrial-antiviral signaling protein (MAVS) (34, 37). Thus, perturbations in normal complosome activities are associated with an increasing range of human diseases, including infections, arthritic diseases, atherosclerosis, and cancer (8).

Therefore, the field has revised its view on complement as a solely plasma-operative system and updated it to one where there is a division of labor where circulating complement guards the vascular space, while local cell-derived canonical complement functions mediate protective tissue immunity and intracellularly active complement orchestrates cell physiology (8)—a concept that could explain the broad impact of complement dysregulation across innate and adaptive immunopathology-mediated human diseases states (14).

## Complement sources in the CNS

Anatomically, the CNS is protected by the skull (and vertebrae), meninges, and cerebrospinal fluid (CSF) and has historically been considered an immune-privileged tissue based on its physical separation from circulation by the BBB, lack of lymphatics, and limited presence of immune cells in the steady-state (45, 46). However, this concept underwent a transformation based on the realization that the isolation of the CNS is not absolute but that it engages into continuous cross talk with the peripheral immune system through the recent re-discovered CNS lymphatic system in the dura mater (47–49). Moreover, classic key mediators of protective (inflammatory) immune responses such as T cells (50) and complement are not only present in the healthy CNS but also contribute to normal brain development and function (51). In fact, the tonic expression of complement in the non-inflamed brain has been noted over three decades ago (52–55) with a broad range of CNS cells being capable of producing distinct complement components.

### Major brain cells

Different types of neurons and glial cells (microglia, astrocytes, and oligodendrocytes) are the major cell types of the brain.

**Figure 2:**
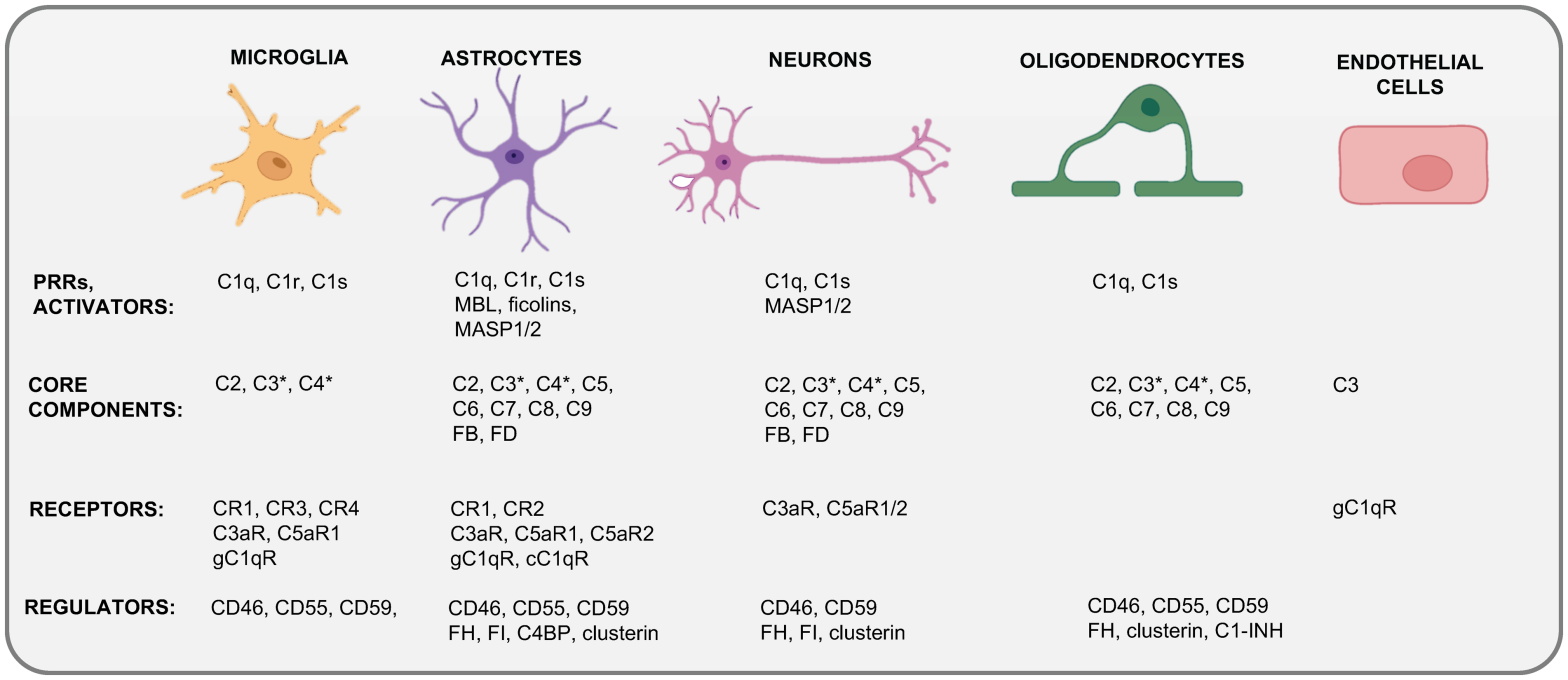
Cellular sources of complement in the CNS. Depicted are the currently known expression profiles of complement system components by major cells in the CNS. Kolmer’s epiplexus macrophages expressing CR1–CR4 and CD55 and CD59 are not shown. This schematic does not discriminate between complement expression measured on the transcriptional (mRNA) or translational (protein) level and combines data derived from resting CNS cells (steady state) and from cells activated during neuroinflammation. C1-INH, C1 inhibitor; C4BP, C4b-binding protein; cC1qR, receptor for the collagen-like region of C1q; CR1–4, “complement receptors 1–4; F, factor; gC1qR, receptor for the globular head region of C1q; MASP1/2, mannose-binding lectin associated protease 1 or 2; MBL, mannose-binding lectin. ^*^C3 and C4 activation fragments can be detected specifically during CNS inflammation or infection and often in proximity to microglia. Created with BioRender.com.

*Microglia*, the brain-resident myeloid cell compartment, perform functions akin to peripheral macrophages in immune surveillance (56) and represent the primary source of complement in the brain (57, 58) (Figure 2). They produce C1q, C3 and C4 (59), and express a broad range of complement receptors and regulators, including C3aR and C5aR1 (60), CR1 to CR4, and CD46, CD55 and CD59 (Figure 1) on their surfaces (54, 55, 61, 62). Similar to peripheral macrophages, microglia respond to stimulation and environmental cues with modulation of their intrinsic complement expression machinery (63). For example, microglia substantially increase C5 production and C5aR1 expression upon peripheral nerve injury (60). And, *vice versa*, stimulation of the C5aR1 on microglia supports their pro-inflammatory effector responses, such as IL-6 and TNF production in a p38 and extracellular signal-regulated kinase (ERK) 1/2 dependent fashion (64).

Astrocytes represent about 30% of cells in the mammalian brain and compose the syncytial networks around neurons. They are essential for normal neuronal activity, provide neurons with the metabolites required for their high levels of energy production and cell function, and lend neurotrophic support by recycling neurotransmitters, fine tuning buffer ion concentrations locally, and interacting with and impacting on the activities of microglia and “incoming” immune cells in the vicinity (65). Human astrocyte-derived cell lines express membrane regulators CD55, CD46 and CD59, as well as the receptors CR1 and CR3, the gC1qR and cC1qRs (66, 67), and C3aR, C5aR1 and C5aR2 (68–70). Astrocytes are further capable of phagocytosis and can subsequently present antigens to adaptive immune cells and therefore contribute to local immune cell behavior [reviewed in (71, 72)]. Because astrocytes are a major source of C3 in the inflamed brain, it is not surprising that astrocyte C3 partakes in the inflammatory milieu-driving astrocyte-microglia axis (73). Microglia-derived C1q induces the generation of neuro-destructive cytotoxic astrocytes (coined A1 astrocytes) (71), which, in turn, cause neuronal cell death by generation of neurotoxic factors, and proinflammatory cytokines, overall perturbing normal dendrite morphology (74, 75). Thus, like microglia, astrocytes express a range of complement proteins in the steady-state, increase their production or expression in response to environmental changes and utilize complement receptor-mediated signals for effector functions.

*Neurons* use chemical and electrical signals to transmit information. They synthesize complement factors both *in vivo* and *in vitro* (58). Neuronal mRNA expression of *C1Q, C2, C3, C4, C5, C6, C7, C8,* and *C9* was detected in control postmortem brain tissue and noted to be increased in AD brain tissue (76). This indicates that neurons can express all complement effector molecules. While less is known about the expression of complement receptors and regulators by neurons [although C3aR and C5aR1 expression has been reported to be induced by IFN-γ-mediated signals (77)], neuronal C3 production is increased during sensory neuron regeneration upon dermal infection-associated tissue injury arguing that neuron-intrinsic complement may also serve specific functions (78).

*Oligodendrocytes* are the myelinating cells of the CNS and have been identified as a significant source of various complement proteins, including C1q, C1s, and C2 – C9 (79), with high C3 protein presence in the resting state. Human oligodendrocyte (HOG) cell lines express complement regulators such as CD46, CD55, and CD59 and secrete complement inhibitors such as C1-inhibitor (C1INH), vitronectin, and clusterin (46). Oligodendrocytes may be susceptible to complement attack-mediated lysis during CNS inflammation [MS and mouse experimental autoimmune encephalomyelitis (EAE)] as they then display a reduction in complement inhibitor expression (46, 80–82).

### Skull, meninges, cerebrospinal fluids, and the blood brain barrier

*The skull* has traditionally been seen as a physical bone shield to protect the brain from mechanical impact or trauma. However, it is now broadly acknowledged that the skull bone marrow (BM) provides a critical local source of monocytes, neutrophils, monocyte-derived macrophages and B cells, which are dispersed into the brain during infection or inflammation (83–87). Further, the composition of skull BM cells is different in the healthy state and in those affected by neurological disorders (88), indicating that the skull BM should be considered as an important player in neuroinflammatory diseases. Moreover, the existence of ossified vascular channels directly connects the skull BM to the dura matter thereby allowing immune cells to migrate independently of the system in circulation. Indeed, a recent study suggests that SARS-CoV2 can infiltrate into the CNS through the skull BM channels, and based on autopsies of COVID-19 patients with long-term neurological complications, it appears that complement activation is among the key pathways induced in the BM by virus presence (89). These findings, in combination with the emerging role for cell-autonomous complement in the development trafficking of BM progenitor cells (90) and the activity of myeloid effector cells (35), strongly suggests the need to study complement in the skull BM in more detail.

*The meninges* are a three-membrane layer system (dura mater, arachnoid mater, and pia mater) located under the skull protecting the brain and spinal cord. They are considered a major immune surveillance organ, as they harbor both innate and adaptive immune cells which provide defense against pathogens but also support immune homeostasis at this location (91, 92). The dura matter contains most of the meningeal immune cells, including neutrophils, (regulatory) T cells, monocytes and macrophages, natural killer (NK) cells, (plasmacytoid) dendritic cells (DCs) (93) and (plasma) B cells (85, 91, 94) and innate lymphoid cells (85, 91, 94). Meningeal immune cells populate the space adjacent to dural venous sinuses, regions of slow blood flow with fenestrations and that can be entry points for bloodborne pathogens to the brain (94); therefore, immune responses within the CNS are often initiated in the meninges before spreading into the parenchyma. There is currently virtually nothing known about the role of complement in meningeal health or inflammation. However, given the widespread presence and functional significance of cell-autonomous complement across immune and non-immune cells, we expect (local) complement to emerge as a critical player at this location as well.

This notion is supported by the finding that the CSF contains several complement proteins, including a broad range of regulators in the healthy host (95). Although a large proportion of CSF proteins can be sourced from plasma, the fact that the CSF circulates through the subarachnoid space of the meninges and disposes of brain molecular waste makes it feasible that complement locally produced by meningeal immune and non-immune cells contributes to the normal CSF complement signature. Bacterial, viral and fungal infections induce a strong increase in CSF complement components, including MBL and C5 which contributes to pathogen control (95, 96). On the other hand, some complement proteins, such as C1q and C3, are pathologically increased in the CSF of patients with MS, where elevated C3 levels correlate significantly with disease progression (97), and in cerebral ischemia (98). Furthermore, the presence of C3/C3a in the CSF has been suggested as a potential biomarker for predicting the prognosis of MS and AD, although the available data is conflicting (99, 100).

The increase in complement proteins in the CSF during CNS infection or neurodegenerative or neuroinflammatory disease is thought to largely be based on the influx of complement proteins from the periphery through a compromised BBB (101). However, the composition of the BBB is complex and includes epithelial and endothelial cells, astrocytes, microglia, and pericytes, and many of these cells express complement proteins that increase in experimental disease conditions (58, 102–105). For example, endothelial cells actively produce C3a which can polarize astrocytes toward an A1 neuro-inflammatory phenotype (106) and engagement of C3aR on the choroid plexus epithelium disrupts the blood-CSF barrier and supports leptomeningeal metastasis into the CSF (107).

Thus, complement contributes to the maintenance of the normal BBB by preventing disease-associated leakage of plasma complement into the CNS but the BBB itself should not be dismissed as a potential source of CSF complement in health and disease. These considerations are not trivial, as they may provide important cues for determining when and where to target complement therapeutically in neurodegenerative and neuroinflammatory diseases. The reality is likely that both local and systemic complement contribute in a spatiotemporal fashion to certain disease states (98).

## Beneficial complement activities in the CNS

**Figure 3:**
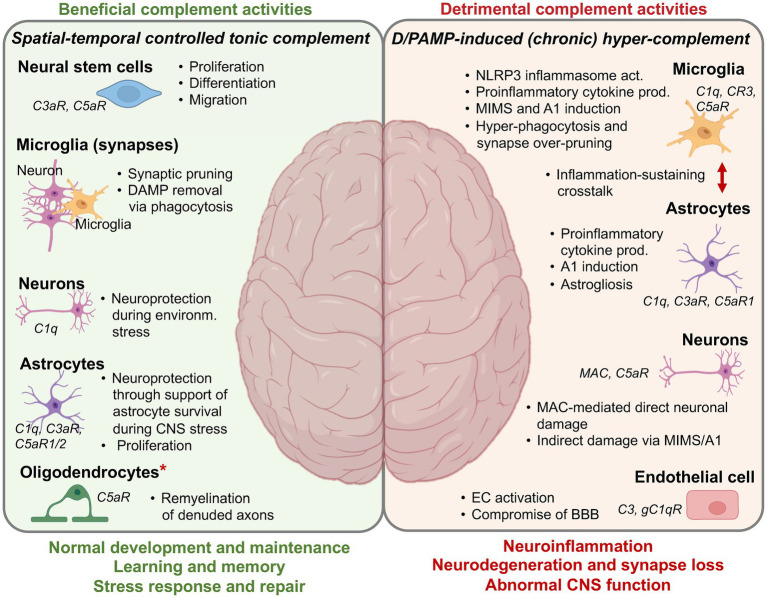
Complement in the CNS—the Good and the Bad. A summary overview over the core beneficial and detrimental activities of complement in the CNS. More detailed information (if available) on the specific molecular mechanisms underlying the depicted complement functions can be found in several recent reviews on this subject matter. A1, neurotoxic, reactive, astrocytes; BBB, blood-brain barrier; DAMP, danger-associated molecular pattern; EC, endothelial cell; MAC, membrane attack complex; MIMS, microglia inflamed in MS; NLRP3, NLR family pyrin domain containing 3. ^*^It is currently unclear if the remyelinating capacity of C5aR1 is due to a direct effect on oligodendrocytes or mediated through an indirect effect on other cells. Created with BioRender.com.

In line with the increasing understanding that complement is not a mere pro-inflammatory immunological weapon but also an integral player in the processes underlying maintenance of tissue homeostasis and repair, it is now apparent that complement is required for normal brain development and function. Beneficial complement activities contribute to normal embryogenesis as well as prenatal and postnatal CNS development through orchestration of brain cell differentiation, neuronal survival, proliferation, migration signaling, and synaptic refinement through pruning and axon myelination (6) (Figure 3).

### Development

Many of the core complement components (for example, C3, C4, and C5) and receptors (C3aR, C1qRs) can be found at particularly high levels in the neural crest during development in developmental models, for example, *Xenopus laevis*. During Xenopus embryological CNS development, neural crest cells must mutually attract through a C3aR-C3a interaction axis and then collectively migrate to form the neural tube. Perturbations in the C3aR-C3a crosstalk results in the loss of neural crest cell organization (108). Complement-mediated cues also guide development of the brain in mice as MASP and C3 knockout mice exhibit clear deficiencies in neuronal migration (46, 109, 110) and abnormal formation of the embryonic neural tube, with varying degrees of severity. Locally produced C5 also partakes in CNS development and support particularly neurogenesis through C5aR1-mediated polarization and proliferation of mouse embryonic neural progenitor cells (111), in addition to providing cues for neuroepithelial cell polarization, an important additional step toward normal neurulation (112). Interestingly, mice deficient in the complement receptor CR2 present with increased basal neurogenesis when compared to wild-type mice (113). Thus, local complement engages important drivers and controllers of neurogenesis and CNS development (114).

### Synaptic pruning

One of the most significant recent discoveries regarding the novel roles of complement in the CNS is the realization that complement functions not only during prenatal stages but also postnatally. Complement components C1q and CR3 play a pivotal role in selectively removing unused or damaged synapses during postnatal brain development, in a physiological process called synaptic pruning (115–117). This developmental phase is characterized by the initial overproduction of neural circuits, followed by synaptic pruning—a process essential for optimizing neural circuitry and enhancing brain connectivity (118). During synaptic pruning, CR3-expressing microglia recognize and eliminate excess or damaged synapses which are marked by C1q deposition and thus display an “eat me” signal (115, 119) and mediate the needed fine-tuning of neural networks post birth (115, 117, 120). The importance of complement in this process has been shown using C3 or CR3 deficient mice, both of which have impaired engulfment of synapses by microglia and exuberant excitatory connectivity in multiple brain regions (116, 121). The process of synaptic pruning is currently considered majorly driven by microglia-mediated phagocytosis, however, an alternative mechanism termed trogocytosis is now receiving increasing attention (122, 123). Complement may also play a role here, as C3 has recently been identified as major driver of human and mouse B cell trogocytosis (124), and the membrane-bound amphibian regulator of complement activation 3 (aRCA3), a homolog of mammalian CD46, mediates axonal pruning in Xenopus (123).

### Myelination

Local complement production also aids in neuron myelination, a complex process involving the formation of myelin sheaths around neuronal axons which is a prerequisite for efficient nerve signal transmission. Oligodendrocytes are central to this process as producers of myelin and mediators of myelin compaction around axons (125, 126). An unexpected key role for C1q in this process was identified through the observation that early C1q deficiency in mouse brains is associated with impaired myelin development *in vivo* (127). Current studies suggests that microglia-derived C1q may contribute to the regulation of oligodendrocyte precursor cell survival and myelination *in vitro* and *in vivo* through a not-yet fully understood molecular mechanism (127) that involves facilitation of myelin protein synthesis, as shown in oligodendrocyte-neuron co-culture (128). Similar to what is known about complement’s role in peripheral tissue regeneration, CNS complement may also support remyelination, the adaptive repair-and-restore response to dys- and demyelination (126). For example, C5 is required for the process of remyelination in chronic lesions of mice in a mouse experimental autoimmune encephalomyelitis (EAE) model, as C5-deficient animals display high gliosis and extensive lesional scarring in combination with reduced axon survival and remyelination when compared to C5-sufficent animals (129). Since C5 is also clearly a driver of CNS pathologies (see below), this finding indicates the complex and context-dependent roles of single complement components in the healthy and diseased CNS.

### Neuroprotection and DAMP clearance

Normally developed and myelinated neurons need to be protected from noxious stimulus-induced damage or death. *In vitro* studies have shown that C1q produced by microglia exerts a direct neuroprotective role against amyloid-beta (Aβ)-induced neurotoxicity through the expression induction of genes encoding proteins associated with neuronal cholesterol metabolism, nerve growth factor (NGF), and neurotrophin-3 (NT-3), which collectively enhance neurite outgrowth. Furthermore, C1q enhances neuronal survival and promotes outgrowth of new neurites (130, 131). Similarly, C5a mediates neuroprotection against glutamate excitotoxicity in an *in vivo* mouse model by retraining the activity of the death caspase-3 in neurons (132, 133), indicating an unexpected novel anti-inflammatory role for C5a in the CNS (133). If cell-protective mechanisms in the CNS fail and extensive cell damage or death occurs, it imperative that these sources of DAMPs are removed rapidly and in a non-inflammatory fashion. The silent removal of apoptotic cells by efferocytosis is key for maintaining CNS homeostasis (134). In the context of efferocytosis, complement proteins serve as “eat me” signals by opsonizing apoptotic cells, thereby tagging them for recognition and uptake by phagocytes (135). Particularly, C1q is upregulated upon CNS injury and during the early stages of neurodegenerative diseases and can then, in line with its major pattern recognition receptor role (Figure 1), initiate removal of apoptotic neurons and neuronal blebs by microglial cells through the initiation of C3b deposition onto the dying cells (136). Furthermore, multiple epidermal growth factor (EGF)-like domains 10 (Megf10), a receptor present in astrocyte membrane, has been shown to bind C1q and mediate clearance of apoptotic cells by astrocytes in the developing cerebellum *in vivo* (137, 138).

Overall, there are several known important roles for complement in normal CNS development and homeostasis, and we fully expect that future studies will define additional housekeeping activities for complement in this tissue.

## Detrimental complement activities in the CNS

Although studies noting increased CNS complement activities in neurodegenerative and neuroinflammatory diseases strongly suggest that augmented complement may contribute to these pathologies (see below for discussion of particular diseases), it was human genetic evidence that ultimately proved this notion to be correct. Large genome-wide association studies (GWAS) performed to identify risk factors for late-onset AD (LOAD) revealed that genes encoding complement receptor CR1 (*CR1*) and the regulator clusterin (*CLU*) were among the significant hits (139, 140). Similarly, exciting recent work identified copy number variations in the genes encoding complement component 4 (*C4A* and *C4B*) as the currently strongest risk factor for the development of schizophrenia (141). In the following we provide some of the key evidence for complement’s contributions to several prevalent neurodegenerative and inflammatory diseases of the CNS.

### Multiple sclerosis

Multiple sclerosis (MS) is a chronic autoimmune disease that affects the central nervous system, including the brain, spinal cord, and optic nerves. It is characterized by the immune system attacking myelin, leading to inflammation (and hence considered largely a neuroinflammatory disease) and damage that typically include the presence of demyelinating lesions in the white matter of the brain and spinal cord, gliosis, and axon damage (142). There are several lines of evidence that suggest involvement of maladaptive complement in the pathogenesis of MS (143, 144). For example, a major feature of sites of active myelin destruction (acute lesions) in the MS brain is the deposition of the C9 neoantigen (indicative of MAC formation), while MAC staining in chronic lesions is less pronounced (145). Furthermore, the numbers of patients with active and chronic active inflammatory demyelinating lesions in the thalamus seemed higher when compared to patients with gray and white matter lesions with complement deposition detected highest in thalamic lesions (146). Postmortem analyses demonstrate non-neuronal and neuronal anti-C3b immunoreactivity (147, 148) and increased *C1QA* gene transcription in neurons in the MS cortical and deep gray matter (149) which may be associated with the IgG aggregate-mediated, complement-dependent neuronal apoptosis observed in *in vitro* studies (150). This indicates that high complement activity is a feature of actively demyelinating (thalamic) lesions while sustained (lower) complement activation associates with chronic lesions. In line with the indication that C1q may be a major player in MS, increased serum C1q levels positively correlate with T2 lesion volume of MS patients (151), and, similarly, augmented C1q protein levels in the serum and brain in mice associate with disease severity in a cuprizone mouse model of MS.

Our lab recently reported profiling of glial and immune cells in samples from patients with progressive MS. Analysis of astrocytes inflamed in MS showed ~20-fold upregulation (relative to nonreactive astrocytes) of genes encoding the C1q complex activators (*C1S* and *C1R*) and C1q receptors (*CALR*, *C1QBP*), as well as upregulation of *C3* (152). Furthermore, we identified C1q as a critical activator of “microglia inflamed in MS” (MIMS), a glial phenotype with neurodegenerative programming. Aligning with these findings, studies in the marmorset EAE model identified C1q-positive axons and neuronal cell bodies in the gray matter bordering immune cells infiltration sites (152), overall suggesting a key role of the CP activator C1q in complement-mediated MS pathology. Interestingly, C1q immunoreactivity in the rhesus EAE model noted cytoplasmic rather than membrane-associated localization indicating local production of C1q, possibly by neurons (153). Effector molecules downstream of C1q and CP activation are also involved in MS (154). For example, increased C4a levels observed in the CSF of patients with MS correlate with disease activity and relapse (155), and C4d has been observed at the borders of intracortical lesions (156). In another study, increased CSF levels of C3a, C4a, and Factor B (FB) cleavage products Ba and Bb were found in MS and correlate with higher levels of disability (154). There is also strong indication that MAC-mediated local cell damage or lysis contributes to MS as systemic inhibition of MAC formation via provision of an antisense oligonucleotide targeting mouse C6 blocked activation of the parenchymal neuroinflammatory responses involving NLRP3 in a mouse EAE model (157).

While the onset of MS is driven by autoimmune and inflammatory responses, disease progression often culminates in neurodegeneration and synapse loss. A recent study suggested a role for C3 in this process, reporting microglial synaptic engulfment and profound synapse loss in MS patients and, in a mouse EAE model, synapse loss independent of local demyelination and neuronal degeneration but coincident with gliosis and increased C3 presence (158). A second, independent, study also noted EAE attenuation in *C3*^−/−^ mice (159). Of note, retinal ganglion cells are also among the cells lost during progression of MS, which causes optic neuritis. Consistent with studies demonstrating that variants in the *C3* gene are associated with accelerated retinal neurodegeneration in human disease, conditional deletion of *C3* in astrocytes in mouse EAE protects against loss of retinal ganglion cells (160). There is increasing indication that activation of the inflammasome, particularly in microglia, is a contributor to their proinflammatory activities in MS (161). Given the emerging strong crosstalk between the (intracellular) complement system and the NLRP3 inflammasome (162), we would argue that this may be a functional relationship that should be further explored with regard to its role in MS pathology.

Although current data are limited, there is indication that local complement activated by self-antibodies to the myelin oligodendrocyte glycoprotein (MOG) may also contribute to acute disseminating encephalomyelitis (ADEM) through cell destruction (163). Moreover, astrocyte death in neuromyelitis optica spectrum disorder (NMOSD) and demyelination in MOG-associated disease (MOGAD), diseases that can mimic MS, involve terminal complement activation and MAC deposition (164–166) as well as C3 activation and neutrophil hyper-activity (167). NMOSD associates with a characteristic pattern of astrocyte dysfunction and loss, resulting in secondary demyelination and neurodegeneration. In most patients, NMOSD is caused by pathogenic, complement-activating, IgG autoantibodies against the main water channel in the CNS, aquaporin 4 (AQP4), and astrocytes are the prime target of the unwanted and detrimental immune response in NMOSD patients. *In vivo*, CNS lesions are characterized by deposition of complement proteins including C1q, C4b, and the MAC (168). The increased levels of soluble MAC (sC5b-9) and the FB activation fragment Ba in the serum of patients in the acute phase of NMOSD strongly correlate with clinical stage. Overall, amplification of initial CP activation by the AP further increases complement activation and contributes to the exacerbation of MAC-mediated NMOSD through C5a generation and recruitment of immune cells (169, 170). The critical involvement of C5-mediated complement effects in NMOSD has been proven unequivocally by the effectiveness of the FDA approved monoclonal antibody therapy targeting C5 for NMOSD (171).

### Alzheimer’s disease

Alzheimer’s disease (AD) is the most frequent form of dementia in the elderly, with approximately 60%–70% prevalence over the age of 80 years. Amyloid plaques and neurofibrillary tangles (NFTs) accumulate (possibly due to age-related reduced efficiency of the cellular debris removal machinery) and sustain neuroinflammation, neuronal and synapse loss and subsequent neurodegeneration as hallmarks of AD (172, 173). A pathological role for complement in AD pathophysiology was suggested by studies in the 1990s, which noted colocalization of complement proteins C1q, C3, and C4 with Aβ plaques in postmortem studies when compared to healthy control tissue (174). Additionally, a clear increase in mRNAs encoding C3 and C4 was noted in the AD compared to the healthy brain (175). An independent study provided evidence that components of the terminal complement pathway (Figure 1) are also present in the brains of AD cases, which seeded the notion that the MAC may contribute to neuronal injury and neurodegeneration (76).

Tangible evidence for a key role of complement in AD was provided in 2009, when two independent GWAS studies identified genes encoding clusterin and CR1 (and later C1S and C9) as significant risk factors to develop AD (139, 140, 176). Also, C4 levels in the CSF of AD patients are modulated in preclinical AD and significantly increase when brain Aβ pathology, tau pathology, and neurodegeneration are measurable (177). A separate recent study observed increased C1q incorporation into extracellular vesicles circulating in the CSF of patents with AD (178). In addition to the postmortem studies, animal models of AD have provided opportunities for mechanistic insights. For example, although global knockout of C1q in the classic beta-amyloid precursor protein (APP) mouse model of AD shows similar Aβ accumulation, the phagocytic capacity of activated forms of microglia was significantly reduced in absence of C1q, and the animals showed less severe neuropathology (179). Downstream of C1q, mice deficient in C3 display accelerate recovery from axon injury compared to control animals (180) and APP/PS1/*C3*^−/−^ mice have better cognitive performance despite similar Aβ level as seen in the control group (181). Moreover, antagonizing C5aR1 signaling on microglia in a mouse model of AD reduces microglia proinflammatory polarization and halts disease progression (182). Similarly, engagement of the C3aR on microglia and neurons fosters neuropathology in a APP model (183).

Overall, it seems that early pathological augmentation in C1q and C3 levels may contribute to synapse loss by undesired over-pruning while downstream complement activation (possibly triggered by C1q) leads to anaphylatoxin receptor-induced proinflammatory programming of microglia, neuronal changes, and local destructive MAC formation. It should be noted though that mouse models particularly involving C3 modulations in AD have provided inconsistent outcomes; for example, an independent study noted accelerated disease in C3-deficient APP mice (184). The reasons are currently not clear but could involve differences in microbiota of animals housed in different facilities and that can have an impact on AD pathogenesis (185) or the usage of global KO animals (see below).

### Parkinson’s disease

Parkinson’s disease (PD) is a degenerative disease involving specifically loss of dopaminergic neurons which leads to progressive motor dysfunction. A diagnosis of PD requires the presence of several neuropathological hallmarks including intracellular protein aggregates called Lewy bodies. Although disease-causing mechanisms are not fully understood, altered mitochondrial activity, abnormal kinase activity, proteasomal and lysosomal dysfunction, and neuroinflammation are among key components in disease pathogenesis (186–189).

A first study in 1980 reported high C3d and C4d deposits within the brains of patients with PD (190). These data were confirmed by subsequent studies that also detected C1q and C7 to C9 in the Lewy bodies (191–193). More recently, several proteomic studies identified complement proteins and apolipoproteins in the plasma of patients with PD as top candidate biomarkers (194, 195). However, another study observed no changes in blood C1q and C3 levels between PD and healthy controls but noted a correlation between levels of C3 and non-motor symptoms in women, highlighting sex-dependent effects of the complement system in different disease and conditions (196). *In vitro* studies in the past attempted to glean insights into mechanistic aspects of complement’s contributions to PD, which may involve the ability of the disease-associated splice variant of alpha-synuclein-112 to initiate complement activation (197). Furthermore, C5a can synergize with IgG isolated from the serum of PD patients to cause death of dopaminergic neurons in rat mesencephalic neuron–glia cultures (198). Work that probes complement in animal models of PD is almost non-existent, indicating a need to better understand the role of complement in PD.

### Huntington’s disease

Huntington’s disease (HD) is a dominantly inherited autosomal disorder caused by the abnormal expansion of three-base-pair (CAG) repeats in the gene encoding huntingtin (*HTT*). The CAG repeats lead to increased polyglutamine modification of the huntingtin protein within neurons and glial cells and eventually to its protein aggregation and dysfunction of affected cells (199). HD symptoms are cognitive defects, motor dysfunction, psychiatric impairments, and subsequent neuronal loss (200). While genetics contribute significantly to HD development in the CNS, recent studies have demonstrated role of immune cells, and particularly monocytes and DCs, in this disease (201–203).

As for many diseases of the CNS, complement activation was observed in the postmortem brain of HD patients: mRNA measurements found ample transcription in microglia and neurons of *C1Q, C1R, C4, C3, CD46, CD55*, and *CD59* in HD patients when compared to healthy controls (204). These results were supported by other studies, including a paper reporting increased expression of *C4A, C4B*, and *C3* genes in different CNS regions of HD patients (205), and augmented expression of C3, C4, C7, and C9 proteins in the CSF, serum, and plasma from HD patients (202, 206). Importantly, elevated levels of C4 and C7 in the CSF were detected before onset of visible HD symptoms, while the level of C9 remained unchanged until an advanced stage of disease (202).

Animal models have provided some understanding of the complement system’s functional involvement in HD. For instance, using a rat model of HD based on by administration of 3-nitropropionic acid (3-NP), which causes striatal degeneration, it was found that administration of C5aR1 inhibitors (PMX53 or PMX205) reduced striatal lesion size, neutrophil infiltration and cell apoptosis (207). Similar data were obtained in a mouse model of HD where C5aR1 inhibition led to a reduction in neuronal death and gliosis and ameliorated disease pathology and behavioral deficits in the mice. How exactly C5aR1 contributes to HD pathology remains to be determined. Of note, deletion of C3 assessed in a transgenic mouse model of HD (R6/2) failed to impact on disease progression or pathology (208). A very recent exciting study reported that the selective loss of synaptic connections in HD patients is associated with C1q-mediated selective elimination of corticostriatal synapses at an early stage in disease pathogenesis (209). Thus, there is accumulation evidence that complement contributes to the pathogenesis of HD, but the complement-mediated molecular pathways engaged remain mostly to be defined.

### Schizophrenia

Schizophrenia is defined as a chronic psychiatric disorder. The disease described by variety of symptoms including delusions, hallucinations, disorganized speech, catatonia, social withdrawal, and blunted affection (210). As for AD, dedicated GWAS studies delivered strong indication that complement is an unexpected but major player in schizophrenia: the extended major histocompatibility complex (xMHC) represents a region of strong association with schizophrenia, and fine mapping of this region then identified copy number variants of the *C4* genes (*C4A* and *C4B*) as the most significant risk factor (211–214). Also, a newly discovered complement C3 and C4 regulator, CUB and Sushi multiple domains 1 (CSMD1), has been placed among the top of genome-wide risk alleles for schizophrenia (213). The FB*F allele may also confer heightened risk for schizophrenia, however, the data so far are inconclusive, with some groups observing an association and others failing to do so (215). In general, GWAS studies have been further supported by reporting increased in complement gene expression, protein concentration, and overall activity in the serum or plasma of schizophrenia cases compared to controls (141, 216, 217). In addition, C3 and C1q protein levels in the blood are significantly increased in patients with schizophrenia when compared to healthy control subjects (218). Given augmented levels of MBL activity in the plasma of schizophrenic patients (216), in combination with the increased presence of C1q, it is likely safe to conclude that, at minimum, LP and CP activation are involved at some level of local complement activation (maybe triggered through changes in immune cell activation or their death)—however, this needs to be formally explored.

An interesting recent study noted that about 20 complement-related genes showed significantly higher expression in the peripheral blood mononuclear cells (PBMCs) of patients with schizophrenia when compared to unaffected individuals (219). This is an important observation because there is accumulating evidence suggesting that at least in a subset of patients, schizophrenia is a neuroimmune disorder. For example, epidemiological studies suggest a correlation between schizophrenia risk and prior diagnosis of autoimmune diseases, previous hospitalization due to infection, and prenatal and childhood infections (220, 221). In addition, clinical studies have shown that inflammatory markers detected in the periphery, such as IL-1β, TNF, IL-6, soluble IL-2 receptor, and C-reactive protein, are elevated in patients with first-episode psychosis and schizophrenia (222). Given the central role of the complosome in the control of immune cell activation, it may be prudent to explore a role for the intracellular complement system in schizophrenia.

As for the mechanism by which C4 may contribute to schizophrenia, Yilmaz and colleagues investigated the role of *C4A* in a humanized mouse model of the disease and found that overexpressing *C4A* reduced cortical synapse density, increased microglial engulfment of synapses, and altered mouse behavior — overall indicating that uncontrolled C4A-mediated synaptic pruning is associated with abnormal brain circuits and behavior (223). Further dissecting the exact, and likely complex, mechanisms by which the complement system initiates/drives schizophrenia pathology will be important.

### Amyotrophic lateral sclerosis

Another CNS disease for which evidence of complement involvement is accumulating is Amyotrophic lateral sclerosis (ALS). Neuroinflammation has been proposed as an underlying mechanism in ALS, and the detection of complement activation fragments in motor neurons and immune cells close to the site of inflammation support this notion. For example, high levels of mRNA encoding *C1QA* and *C3*, *C4* and components of the MAC are noted in the spinal cord and motor cortex of patients with ALS (224). Furthermore, increased presence of complement proteins in the CSF defined samples from patients with fast-progressing ALS vs. those with slow-progressing disease (225). These data, together with the finding that the pharmacological inhibition of C5aR1 signaling in a mouse SOD1 (G93A) disease model of ALS ameliorates disease pathology (226), strongly indicate that (local) complement activation may be a contributing factor to this debilitating neurodegenerative condition and should be explored for future therapeutic targeting.

## Emerging concepts and considerations

Although there is evidence that plasma-derived complement that can enter the brain through a compromised BBB during neuroinflammation, it is generally accepted that local production is a major driver of the beneficial and detrimental effects of complement in the CNS. Perturbations of the complosome have been associated with a range of diseases but a potential role in brain development, normal function, and/or neurodegenerative and neuroinflammatory diseases has so far not been explored. Given the particularly intimate functional relationship between the complosome, mitochondrial biology, and cell metabolism and the emerging concept that pathological metabolic changes and mitochondrial dysfunction in microglia and brain-infiltrating macrophages contribute to smoldering inflammation in neuroinflammatory diseases (227), we argue that the complosome is a likely player in CNS pathologies. For example, CD46 controls the balance of arginine vs. glutamine flux in T cells (228), and this pathway could possibly extend to neurons, which require arginine for survival and function. In addition, heightened intracellular C5 activation in human and mouse macrophages induces mitochondrial reactive oxygen species (ROS) production and an inflammatory phenotype in these cells (35), a scenario that may also be applicable to chronically activated microglia. We therefore suggest that modulations in complosome activities should be considered in future studies on CNS pathologies. We do acknowledge that this will be not easy from a technical point of view. However, to date, complement-targeting drugs in the clinic are aiming at circulating complement and will likely not be effective in tissue or intracellular complement-driven disease states. Thus, an investment in understanding the functions and control mechanisms of tissue- and cell-autonomous complement may be important to develop improved drugs for common diseases.

Another area of interest to watch may be a potential association between viral infections, neurodegeneration, and complement (229). A substantial number of CNS-tropic viruses, including herpes virus 1 (HSV-1), herpesvirus 6 (HHV-6), human immunodeficiency virus (HIV), and measles virus (MV) have been shown to induce complement activation as an anti-pathogenic response in the brain [for an excellent recent review, see (229)] and latent HSV-1 infection may be associated with increased risk for AD (230). Moreover, MS and AD patients show an over-proportional presence of HHV-6A and HHV-6B infections (231, 232). These infections also accelerate disease course in a marmoset EAE model of MS (233). Epstein Barr virus (EBV) establishes life-long latency in the host, and is increasingly implicated as a major factor in the causal chain of both MS (234) and potentially AD (235). These latter findings are particularly interesting as EBV utilizes CR2 (CD21) as cell-entry receptor (236), which opens the possibility that virus-induced changes in CR2-mediated signaling of infected cells may be a contributing factor in addition to virus-triggered local complement activation. Similarly, MV infection, which can cause complications including encephalomyelitis (237) engages the complement regulator and C3b/C4b receptor CD46 on microglia to induce cell–cell fusion in the brain and thereby viral spread (238). The mechanisms underlying pathogen-initiated CNS complement activation and neurodegeneration are not understood but could involve unwanted pruning of “bystander” synapses. With regard to cell-autonomous complement activity, our collaborators have recently shown that C5 produced by macrophages is critically required to control *Candida albicans* infections in susceptible individuals through C5a-mediated metabolic programming, which enables macrophages to engage in candida clearance (239). Overall, a better understanding of the mechanistic links between CNS infection, complement, and neurodegenerative diseases may provide new insights that can eventually be harnessed therapeutically.

Finally, animal models involving complement deficiencies have been immensely helpful in dissecting complement-mediated pathological contributions to neurodegenerative and neuroinflammatory diseases and have served as pioneering platform for *in vivo* testing of complement inhibitors (240). However, most studies on complement in CNS pathologies have been performed using mice with global deficiencies in complement components of interest. Given that most complement receptors, and particularly C3aR and C5aR1 and C5aR2 function in a cell-specific and often temporal fashion, it may be prudent to assess mice with (brain) cell-specific deletions for consistency with previous observations. Furthermore, there are species-specific important differences in complement genes and pathways that may make interpretation of mouse-derived data not straightforward. For example, mice lack CD46 expression on somatic tissues (241) and harbor two *CD59* genes instead of one as observed in humans (242), and humans express *CR1* and *CR2* as single genes, whereas mice express a *CR1/CR2* hybrid gene (243). In addition, there is notable divergence in the complement gene module between rodent and primate microglia (244). Thus, it will be important to assess for transferability of result derived from specific complement mouse models to the human disease pathology.

Despite these open questions, the large body of evidence reviewed here provides strong support for the continued investigation of the multifaceted roles of complement in neurodegenerative and neuroinflammatory diseases and provides hope that modulation of complement pathways can be broadly exploited in future treatment of those disorders.

## Author contributions

LN-D: Conceptualization, Data curation, Investigation, Writing – original draft, Writing – review & editing. AM: Conceptualization, Data curation, Investigation, Writing – original draft, Writing – review & editing. DR: Conceptualization, Data curation, Funding acquisition, Writing – original draft, Writing – review & editing. CK: Conceptualization, Funding acquisition, Investigation, Visualization, Writing – original draft, Writing – review & editing.
